# Supplementary material for: Patterns of Intron Gain and Loss in Fungi
Source: PLoS Biol. 2004 Nov 30;2(12):e422. doi: 10.1371/journal.pbio.0020422 (PMC532390; doi:10.1371/journal.pbio.0020422)
Supplement: Table S1 — Also available at http://genes.mit.edu/NielsenEtAl/. (4.3 MB ZIP). [file pbio.0020422.st001.zip › NielsenEtAl/html/22.html]

AN3745.1.NCU08121.1.MG09302.1.FG07107.1


```
 CLUSTAL W (1.82) Multiple Sequence Alignments - Introns Inserted


Sequence 1: NCU08121.1	200 aa
Sequence 2: FG07107.1	191 aa
Sequence 3: MG09302.1	193 aa
Sequence 4: AN3745.1	218 aa
Alignment Length: 221 aa
Number Identitical Residues: 85 aa
Alignment Score (without introns) 4197


MG09302.1 	--------MSSAKPQA-KL~PVAVNKPTPYSFDLA1LLLANDPNPVELDKT-DLEGSLSS
NCU08121.1	-----MSDATTTTTTT-KL~PVTVDKPTPYTFDLG~LLLANDPNPLLLDRSSALEPQLAS
FG07107.1 	--------MSLPNSKP-KL~PVAVEKPTPYTFDLG~HLLAEDPNPVTLDRE-NLEQSLAE
AN3745.1  	MANSETEAKSTIKPKPERL2PITVSKPTPYTFDLG~HLLANDPNPLELPKSEPLNASLKA
          	 :.:. .  :  .. ..:* *::*.*****:***.  ***:****: * : . *: .*  

MG09302.1 	LARDGAQALINQLLTTCPIQSTPSGVLLSLPAQQTALPREKPLPAAKEPTKWERFAAKKG
NCU08121.1	IARDGCQSLINQLLTACPISSTPAGVLLSLPPPQTPLPREKPVPTPKAETKWSQFAKRRG
FG07107.1 	LARDGAQSLINQFLTTCPLSSTAEGVLLTLPAPSTRLPREKPVPQAKAPTKWERFAAKKG
AN3745.1  	TARDGVQSLLNQLLTTCPITSSQQGVLLTLPAPSTILPRHKPLPTPKPPTKWELFARKKG
          	 **** *:*:**:**:**: *:  ****:**. .* ***.**:* .*  ***. ** ::*

MG09302.1 	I-----KP----KTREQRRNIQYNEEKGEWERKWGYKGANKQLEKDWLVEVDPA---KEA
NCU08121.1	I-----KP----KTREQRRNLQYDETTGEFGRKWGYKGANKAGEDAPIIEVKKS---QEE
FG07107.1 	I-----KP----KTRESRRNLAFDEQTGEWQRKWGYKALNKKGEDWPIVEVDME---AEK
AN3745.1  	IGKYSNKPGAALADKERRKKLVYDEESGEWVPRWGYKGKNKD-DDQWLVEVKEKDWKKEE
          	*.. :.**.::   :* *::: ::* .**:  :****. **  :.  ::**.  . . * 

MG09302.1 	KLK-EGETLRGQTRRERVERQKRNERKMRKNER--DAAKK---
NCU08121.1	KRMAEGKSVRGDKRREIRERVKRNERKMRANERRESGRKK---
FG07107.1 	KRK-EGTTIRGDGRRERKERVKRNERLMRKNK----GIPK---
AN3745.1  	EAAAKGSSIRGMSRAERKERIRRNERKMRANER--NSRKSGGK
          	:  ::* ::**  * *  ** :**** ** *:   ..  ....
```
